# Differences in seasonal survival suggest species‐specific reactions to climate change in two sympatric bat species

**DOI:** 10.1002/ece3.5292

**Published:** 2019-07-02

**Authors:** Christine Reusch, Jutta Gampe, Alexander Scheuerlein, Frauke Meier, Lena Grosche, Gerald Kerth

**Affiliations:** ^1^ Applied Zoology and Nature Conservation, Zoological Institute and Museum University of Greifswald Greifswald Germany; ^2^ Max Planck Institute for Demographic Research (MPIDR) Rostock Germany; ^3^ Echolot – Büro für Fledermauskunde Landschaftsökologie und Umweltbildung Münster Germany

**Keywords:** hibernation, *Myotis daubentonii*, *Myotis nattereri*, seasonal survival, summer and winter survival

## Abstract

Long‐lived animals with a low annual reproductive output need a long time to recover from population crashes and are, thus, likely to face high extinction risk, if the current global environmental change will increase mortality rates. To aid conservation of those species, knowledge on the variability of mortality rates is essential. Unfortunately, however, individual‐based multiyear data sets that are required for that have only rarely been collected for free‐ranging long‐lived mammals. Here, we used a five‐year data set comprising activity data of 1,445 RFID‐tagged individuals of two long‐lived temperate zone bat species, Natterer's bats (*Myotis nattereri*) and Daubenton's bats (*Myotis daubentonii*), at their joint hibernaculum. Both species are listed as being of high conservation interest by the European Habitats Directive. Applying mixed‐effects logistic regression, we explored seasonal survival differences in these two species which differ in foraging strategy and phenology. In both species, survival over the first winter of an individual's life was much lower than survival over subsequent winters. Focussing on adults only, seasonal survival patterns were largely consistent with higher winter and lower summer survival but varied in its level across years in both species. Our analyses, furthermore, highlight the importance of species‐specific time periods for survival. Daubenton's bats showed a much stronger difference in survival between the two seasons than Natterer's bats. In one exceptional winter, the population of Natterer's bats crashed, while the survival of Daubenton's bats declined only moderately. While our results confirm the general seasonal survival pattern typical for hibernating mammals with higher winter than summer survival, they also show that this pattern can be reversed under particular conditions. Overall, our study points toward a high importance of specific time periods for population dynamics and suggests species‐, population‐, and age class‐specific responses to global climate change.

## 1 INTRODUCTION

Seasonality describes a regularly and often predictable change in the environment following an annual cycle (Battey, 2000). In regions showing strong seasonality, organisms developed various mechanisms such as migration (Gienapp, 2012) and hibernation (Geiser, 2013) that allows them to survive seasons with food and/or water shortages. Especially in times of climate change, it is crucial to understand the impact of changing environmental conditions on the abovementioned mechanisms to deal with seasonality, as this may strongly effect individual survival and, thus, ultimately population persistence (Newson et al., 2009; Sherwin, Montgomery, & Lundy, 2013). As a first step, it is necessary to identify basic annual survival patterns and assess their variability across years. The identification of seasons that are potentially sensitive to environmental changes and the assessment of their importance for individual survival, as well as the identification of individual traits (e.g., sex, age class) that influence survival outcomes, are crucial for understanding current and future population dynamics (Le Cœur, Chantepie, Pisanu, Chapuis, & Robert, 2016).

Seasonal survival is comparatively well studied in birds, due to the availability of long‐term studies of marked populations, high‐quality long‐term ringing data, and sophisticated statistical methods for survival analysis (Klaassen et al., 2014; Leyrer et al., 2013; Rockwell et al., 2017). In this field of research, the concept of summer‐ and winter‐regulated populations has been introduced some time ago (Blumstein & Fernández‐Juricic, 2010; Newton, 1998). This concept states that in smaller species, populations tend to be regulated by high mortality during winter, while particularly in larger species, reproductive rates and high mortality during the breeding season determine population dynamics (Blumstein & Fernández‐Juricic, 2010; Newton, 1998). This pattern, however, breaks down when hibernating mammals are considered. Hibernation is a strategy to overcome times of food or water shortage by means of a reduction in body temperature and thus energy consumption (Geiser, 2013) and allows retirement into microhabitats with clement climate, such as burrows or caves. This special adaptation might enable even smaller‐sized species to reach low mortality levels during winter, counter to the above expectation. So far, only few studies were able to quantify seasonal variation in individual‐based mortality in long‐lived mammals, due to a paucity of suitable long‐term data sets (Fleischer, Gampe, Scheuerlein, & Kerth, 2017; Marra, Cohen, Loss, Rutter, & Tonra, 2015; Turbill, Bieber, & Ruf, 2011). The available studies, indeed, suggest that in hibernating animals, winter mortality does not exceed or is even lower than mortality during summer breeding season (Fleischer et al., 2017; Turbill et al., 2011).

In comparison with other mammals of a similar body size, hibernating bats are exceptionally long‐lived (Munshi‐South & Wilkinson, 2010; Wilkinson & Munshi‐South, 2002). With their low annual reproductive output (Linton & Macdonald, 2018), bats’ population dynamics are driven mainly by adult mortality (Fleischer et al., 2017). Consequently, information on survival of hibernating bats which are, as bats in general, of high conservation concern (Habitats Directive 92/43/EEC, 1992) is of particular importance in times of global climate change with an expected increase in temperatures, changed precipitation patterns, and a higher frequency of extreme weather events, which all may affect seasons differently (IPCC, 2013).

Vespertilionid bats from the temperate zone are renowned for their ability to hibernate using torpor (Kunz, 1982). Despite several studies (Bezem, Sluiter, & J.W. & Van Heerdt, P.F., 1960; Lentini, Bird, Griffiths, Godinho, & Wintle, 2015; O'Shea, Ellison, & Stanley, 2004), there is still a paucity of individual‐based studies on survival rates in bats (O'Shea et al., 2004) that might be able to provide a baseline for mortality. One of the first studies in bats that aimed to estimate survival was done in the 1950th, and soon afterward, several studies investigated survival based on banding thousands of bats captured at hibernacula in the Netherlands (Bezem et al., 1960; O'Shea et al., 2004). Nevertheless, in case of survival studies the banding itself can impact the results by decreasing survival through injurious banding or disturbance in sensitive phases (e.g. pregnancy or hibernation) due to repeated handling to identify the ring numbers (O'Shea et al., 2004). An alternative approach is the usage of RFID tags that allows continuous and automatic monitoring of individuals at the study sites (Gibbons & Andrews, 2004; Kerth & König, 1999; O'Shea et al., 2004). The advantages of the method are individual recognition, a low loss rate of RFID tags over time, reduced disturbance after an initial capture and marking of the individual, and high recapture rates without the need to directly handle individuals (Gibbons & Andrews, 2004; Kerth, Perony, & Schweitzer, 2011; O'Shea et al., 2004).

Exemplary studies that tried to quantify mortality during hibernation in bats suggested a low winter mortality or at least one that is comparable to mortality during summer (Culina, Linton, & Macdonald, 2017; Fleischer et al., 2017; Fritze & Puechmaille, 2018; Sendor & Simon, 2003). However, these previous studies used different methods and sampling strategies, and their data are not easily comparable. Fritze and Puechmaille (2018) for example described very low baseline mortality during hibernation based on dead bat counts in hibernacula. The authors explain the low number of dead bats found in hibernacula either due to a high rate of survival during hibernation or that sampling once per year might not be suitable to discover the real mortality rate (Fritze & Puechmaille, 2018). In a different approach, Culina et al. (2017) used multistate capture–mark–recapture models based on ringing data of three different bat species at their summer roosts, covering seven years to show the effect of individual traits and selected weather parameters on survival. The authors found differences in survival across species and generally lower winter survival of juveniles (Culina et al., 2017). In again a different approach, Fleischer et al. (2017) used a large individualized data set spanning 19 years. This data set was based on summer records of Bechstein's bats (*Myotis bechsteinii*) that had been marked with individual RFID tags, in early and late summer. This study revealed that both summer and winter mortality is equally low in most years (≤10%) but that extreme mortality (>50%) occurred during one winter (Fleischer et al., 2017).

In this study, we used a large individualized multiyear data set collected at a RFID‐monitored hibernaculum to investigate survival in two sympatric European bat species over five years. Thus, we had the valuable advantage (Clutton‐Brock & Sheldon, 2010) of a multiyear data set with high recapture rates without disturbing the individuals unnecessarily that allowed us to account for individual variation in survival and included both sexes and different age classes. Our aim was to identify potential differences in the seasonal survival patterns of two sympatric bat species that live in the same environment during the hibernation period. Our two study species, Natterer's bats (*Myotis nattereri*, further on also referred to as mn) and Daubenton's bats (*Myotis daubentonii*, further on also referred to as md), hibernate in the same hibernaculum in North Rhine‐Westphalia (Stumpf et al., 2017; Trappmann, 2005). Both species are very long‐lived (maximum life‐span > 20 years) (Wilkinson & Munshi‐South, 2002) with females having one offspring per year at most (Linton & Macdonald, 2018). Despite their similar life history, the two species differ with respect to foraging strategy and activity pattern during the hibernation period. Daubenton's bats hunt above water, and there is no evidence for foraging during winter (Flavin, Biggane, Shiel, Smiddy, & Fairley, 2001; Kokurewicz, 2004; Siemers, Dietz, Nill, & Schnitzler, 2001). In contrast, Natterer's bats glean arthropods from surfaces (Andreas, Reiter, & Benda, 2012; Siemers, Kriner, Kaipf, Simon, & Greif, 2012; Siemers & Schnitzler, 2000) and radio‐tracking data suggest that they feed during hibernation period (Hope et al., 2014). Due to high site fidelity with respect to hibernacula in both of our study species (Steffens, Zöphel, & Brockmann, 2004), we were able to follow individuals over a substantial part of their life and to quantify seasonal survival over several years.

As in other studies on bats, we expected lower survival of bats during the first winter while adult bats were expected to have high survival rates during winter (Culina et al., 2017; Sendor & Simon, 2003). We predicted differences in survival across adults of both species due to differences in hibernation strategy. As a consequence of their higher activity during the hibernation period, Natterer's bats are supposed to be highly sensitive to adverse weather conditions that result in low arthropod availability during winter. Because Daubenton's bats do not hunt during the hibernation period, they should be less affected by adverse weather conditions prevailing then. Instead, for successful hibernation, they rely entirely on the fat reserves built up during summer. Thus, Daubenton's bats should depend more strongly than Natterer's bats on good conditions (high arthropod availability) during summer to refill their energy reserves after hibernation and further on accumulate fat reserves for the subsequent hibernation period. As a consequence of the species‐specific demands, we expected differences in the seasonal survival pattern between Daubenton's bats and Natterer's bats.

## 2 MATERIAL AND METHODS

### 2.1 Bat capture and data logging

To investigate survival and the factors influencing it, we used data of 820 individually marked Natterer's bats and 625 individually marked Daubenton's bats at the hibernaculum “Brunnen Meyer,” an old well shaft within a small, permanently for bats accessible house, in North Rhine‐Westphalia (Stumpf et al., 2017; Trappmann, 2005), which was continuously monitored. By positioning harp traps at the entrance of the well house on multiple nights between 15th of August and 1st of October each year (Table S1), Natterer's bats were captured and RFID‐tagged (ID‐100, Trovan) since 2002, Daubenton's bats since 2008. Age class (juvenile or adult) and sex were recorded for each individual (Brunet‐Rossinni & Wilkinson, 2009). Juveniles were distinguished from adults under careful consideration of several age characteristics (e.g., coloration of chin spots, epiphyseal closure, level of dental calculus and tooth abrasion, as well as the lack of signs of reproduction; Brunet‐Rossinni & Wilkinson, 2009; Racey, 1974; Richardson, 1994). If we were not able to identify an individual as juvenile without a doubt, it was treated as adult. All handling and marking of the bats were conducted under the permits for species protection issued by the nature conservation authority of the district Coesfeld (70.2.2.27, 70.2‐0197/08, 70.2‐0228/10, and 70.2‐2012/0254). A RFID‐logger antenna system (LID‐650, EURO I.D.) was positioned at the two small entrances of the hibernaculum. This allowed us to record continuously the unique transponder ID as well as the date and time of passing of tagged individuals during five hibernation periods (2010/2011‐2014/2015) without disturbing the bats (Kerth & König, 1999; Kerth et al., 2011).

Since the presence of only one antenna per entrance did not allow us to directly distinguish between bats entering and exiting the hibernaculum, we separated the hibernation period (August–April) into an arrival period (August–December) and a departure period (January–April) based on activity patterns known from direct observations in the field, light barrier recordings, and the available RFID recordings (L. Grosche and F. Meier, unpublished data). Because of the bats’ very high fidelity to their hibernaculum (Steffens et al., 2004), individuals were considered dead if they were not recorded within an entire hibernation period. Survival was coded as 0 if the individual was considered dead and as 1 if it was known to be alive. We cannot absolutely exclude emigration, but note that in our 5‐year study period of continuous monitoring at the hibernaculum “Brunnen Meyer,” only one out of 1,445 tagged individuals re‐occurred after not being recorded for a complete hibernation period. Additionally, in 13 years of bat surveillance only 24 out of 1,111 bats assigned dead according to our criteria re‐appeared at another RFID‐monitored hibernaculum in close proximity to the study site for at least one more year (L. Grosche and F. Meier, unpublished data). Nevertheless, to assure that individuals have indeed used the hibernaculum, we excluded those individuals from the analyses that had not been recorded either during the arrival or during the departure period of a given hibernation period, but re‐appeared later (mn: 62 out of 820 individuals (7.5%); md: 24 out of 625 individuals (3.8%), Table S2).

Individuals, for whom the last recording was during an arrival period (but not again thereafter), consequently had survived the preceding summer but were assumed to have died in the following winter. Similarly, those bats that were recorded for the last time during a departure period had survived the preceding winter but were assumed to have died during the following summer period. In our study, “winter” (arrival at hibernaculum August–December) includes, depending on the species and sex, a more or less intensive autumn swarming period at the hibernaculum, and the hibernation itself, while “summer” (emergence from hibernaculum January‐April) includes the transfer flights from and to the hibernaculum, as well as the time in their summer habitat.

### 2.2 Statistical analyses

As a first exploratory step of our analysis, the proportions of survivors in the two species were compared in each of the 10 periods (two seasons—summer and winter—in five years, from winter 2010/11 to summer 2015). We tested the hypothesis of equal survival in the two species (two‐sided alternative, chi‐square test statistic with *df* = 1, Yates’ continuity correction) using the function prop.test in R (R development Core Team, 2015).

Regression models were then used to study the effects of the individual characteristics sex and age class as well as of changing environment on survival. The binary response—survival or death—was modeled by logistic regression with an individual‐specific random intercept to account for repeated observations per individual and potential interindividual variation in survival which remained unexplained by the covariates. As Culina and co‐authors in their study on three bat species reported differences between sexes and age classes, as well as different effects of environmental parameters on survival (Culina et al., 2017), we set up the model separately for each species. Sex and age class (juvenile or adult) were included as binary covariates with fixed effects. As the animals were observed at the hibernaculum, it should be noted that survival of juveniles can only be estimated in their first winter; thereafter, the animals enter the adult age class (Table S3).

To capture the impact of changing environmental conditions over the study period, models of increasing complexity were estimated. The simplest model included the binary covariate season (summer vs. winter), postulating a stable seasonal pattern in survival, but no interannual variation. The next more complex model allowed that each year could have its own level of mortality (categorical covariate with one level for each year) combined with an additive effect of season. This model describes a consistent summer‐to‐winter difference (on the logit scale) in survival, but on possibly different levels across years. The most flexible (and least parsimonious) model included a covariate that permits a different level of survival in each of the periods and forces no constant summer‐to‐winter relation. Note that this final model is formally equivalent to the model including a year‐by‐season interaction, but estimates are easier to interpret when coded as a single variable with a level for each period.

We compared models based on the values of the Akaike information criterion corrected for small sample sizes (AICc) (Burnham, Anderson, & Huyvaert, 2011; Hurvich & Tsai, 1991), which converges to the AIC for large samples. Simpler models were selected over more complex models with a lower AICc whenever the differences in the AICc values were smaller than 2 (Burnham et al., 2011; Culina et al., 2017). Furthermore, we calculated the intraclass correlation coefficient (ICC) on the scale of the linear predictor from the variance of the individual‐specific random intercept (Rodríguez & Elo, 2003). All models (generalized linear mixed models) were fitted using R, version 3.4.0 (library lme4, function glmer) (Bates, Mächler, Bolker, & Walker, 2015; R Development Core Team, 2015).

## 3 RESULTS

### 3.1 Differences in survival between the two bat species across periods

**Figure 1:**
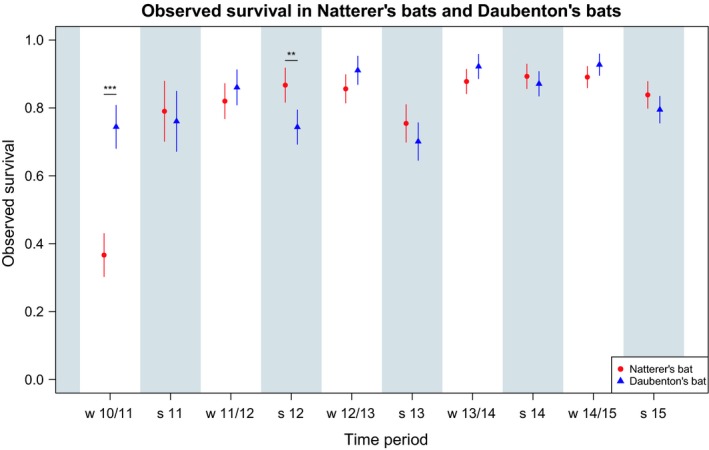
Comparison of observed survival between species (Natterer's bats (*Myotis nattereri*, red points); Daubenton's bats (*Myotis daubentonii*, blue triangles)) in each period. Winter periods are denoted by “w” and the respective years, while summer periods are labeled as “s” combined with the respective year. Additionally, periods that describe summer survival are indicated by a light gray background. Vertical lines indicate 95% confidence intervals for the estimated survival probabilities. The stars in winter 2010/11 and summer 2012 indicate significant differences in observed survival (adults and juveniles combined) between species, see Table S4

Significant differences in survival between the two species were only found in two periods: winter 2010/2011 and summer 2012 (Table S4; Figure 1). While in winter 2010/2011 Natterer's bats showed a much lower survival than Daubenton's bats, in summer 2012 a contrasting pattern was detected. Otherwise, both species showed similar survival. Furthermore, a comparatively low survival in summer 2013 was similarly observed for both species. The observed differences in survival between species in the abovementioned periods indicated a potentially species‐specific sensitivity to certain environmental events and their timing.

### 3.2 The exceptional winter 2010/11

Winter 2010/11 evidently was an exceptional period for Natterer's bats (Figure 1), and including this winter in the regression model inevitably would force the choice of the least parsimonious model, no matter whether the rest of the study period would allow a simpler model. We therefore built the regression model for this species from summer 2011 to summer 2015, excluding the exceptional winter 2010/11.

### 3.3 Importance of individual characteristics

**Figure 2:**
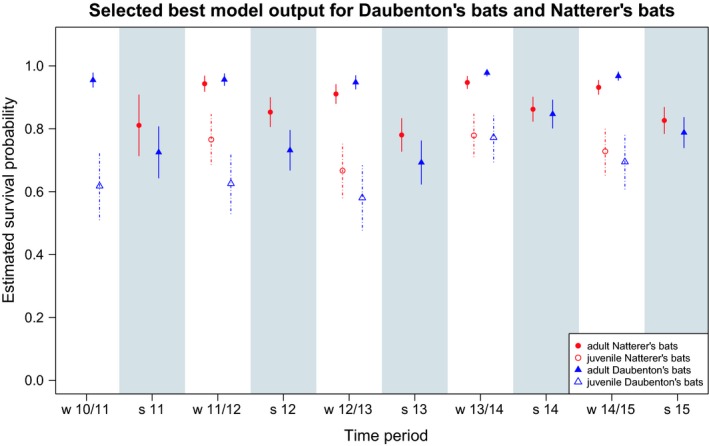
Estimated survival probabilities based on the finally selected mixed‐effects logistic regression models. In both species‐specific models, a random intercept was included for each individual. The model for Natterer's bats (*Myotis nattereri*, red circles/points) was built excluding the exceptional winter 2010/2011. The results of the best model for Daubenton's bats (*Myotis daubentonii*, blue triangles) and of the one for Natterer's bats—in both cases including season, age class, and year as fixed effects— are shown. Winter periods are denoted by “w” and the respective years, while summer periods are labeled as “s” combined with the respective year. Due to sampling at a hibernaculum, we were only able to estimate juvenile survival over winter. Juveniles are characterized by open symbols, while adults are defined by filled symbols

As predicted, age class was a major determinant of survival in both species. Survival of juveniles during their first winter was on average about 30 percentage points lower in Daubenton's bats and 20 percentage points lower in Natterer's bats (Table S5; Figure 2). Neither of the species‐specific models supported a general sex difference in survival (Table 1). The standard deviation of the individual‐specific random intercept was comparable in both species, leading to a moderate intraclass correlation (ICC) of about 0.2 (Table S5).

**Table 1:** Model selection to choose the best fitting fixed effect structure for Natterer's bats (*Myotis nattereri*), top, and for Daubenton's bats (*Myotis daubentonii*), bottom

Model	*K*	AIC_c_	dAIC_c_
Natterer's bats (*Myotis nattereri*)			
age	3	1,852.96	48.15
age + sex	4	1,854.33	49.52
age + season	4	1,809.06	4.26
age + season + year	**8**	**1,806.74**	**1.94**
age + period	11	1,804.80	0.00
age + sex + period	12	1,806.21	1.41
Daubenton's bats (*Myotis daubentonii*)			
age	3	1,886.23	171.13
age + sex	4	1,888.21	173.11
age + season	4	1,727.57	12.47
age + season + year	**8**	**1,715.10**	**0.00**
age + period	12	1,717.65	2.56
age + sex + period	13	1,719.64	4.54

Note

A random intercept was included for each individual. Model selection was based on the AICc. Smaller values of the AICc are preferred, but the difference dAICc in AICc values has to exceed 2 so that a more complex model is selected over a simpler one. The finally selected model is printed in bold.

### 3.4 The effects of season and year

Following the model selection criteria outlined above, the final selected model was the same for both species (Table 1). Besides age class, season and year were both included as predictors of survival. For Natterer's bats, the model with an interaction between season and year returned a smaller AICc. However, this did not exceed the required difference of 2 to the next best and simpler model. Thus, adult survival in both species showed a consistent pattern between seasons (summer vs. winter) that varied in severity across years.

For adults, survival during winter was significantly higher than during summer in both species. However, adult Natterer's bats consistently had higher survival rates compared with Daubenton's bats during summer, but had lower survival rates than Daubenton's bats during winter (Table S5; Figure 2). So, the differences between summer and winter mortality were more pronounced in Daubenton's bats. Finally, juveniles in their first winter suffered from mortality that reduced their survival chances to levels even lower than the species’ summer survival.

Excluding the exceptional winter 2010/11 in Natterer's bats, differences in survival across years, relative to the average level, were smaller than the winter–summer contrast in both species. In particular, the year 2012/2013 resulted in lower adult survival in both species, while the year 2013/14 fostered above average survival in both species (Table S5; Figure 2).

## 4 DISCUSSION

### 4.1 Differences in survival between the two bat species across years

In accordance with the known longevity of both of our study species (Dietz, Nill, & Helversen, 2016; Wilkinson & Munshi‐South, 2002), we found no consistent difference in observed survival between Natterer's bats (range in the different periods: 0.37–0.89) and Daubenton's bats (0.70–0.93) (Figure 1), which was also evident in the estimated survival (Figure 2). However, there were specific periods, namely winter 2010/2011 and summer 2012, in which the two species showed significant differences in survival. Both species had lower survival in the winter 2010/2011, but only Natterer's bats experienced a pronounced population crash during this winter with a survival of only 0.37 compared with 0.75–0.89 in the other periods. In southern Germany, Fleischer et al. (2017) found that Bechstein's bats populations also crashed in the winter 2010/2011. The authors showed that the population dynamics in their 19‐year data set was driven by this single winter and concluded that rare catastrophic events have a major influence on population dynamics (Fleischer et al., 2017). Our data suggest that this may also be the case for other bat species, albeit the pattern seems to be species‐specific as shown for Daubenton's bats that have been much less impacted by the winter 2010/2011, even though they used the same hibernaculum as the Natterer's bats.

A potential cause for these differences in the importance of specific periods for survival might be a species‐specific influence of weather conditions at different times of the year. Depending on the timing of unfavorable conditions and consequently low arthropod availability, bat species may be affected differently due to their distinctive foraging strategies and activity patterns (Culina et al., 2017; Dietz et al., 2016; Siemers et al., 2001, 2012; Siemers & Schnitzler, 2000). Robinson and co‐authors found in seven out of ten bird species a positive correlation between survival and the North Atlantic Oscillation index (NAO, December–March) with strong positive effects in four species (Robinson, Baillie, & Crick, 2007). The NAO, which is based on the difference of normalized sea level pressure between Lisbon, Portugal and Reykjavik, Iceland (Hurrell, 1995), is described as a large‐scaled weather parameter for winter severity with negative values indicating cold and dry winters (Hurrell, 1995; Post, Stenseth, Langvatn, & Fromentin, 1997). Interestingly, the winter 2010/2011 with an exceptionally low survival in Natterer's bats was one of the two winters in our study period that had negative NAO values (winter 2010/2011: −1.57; winter 2012/2013: −1.97 [Figure S1]; data were provided by the Climate Analysis Section, NCAR, Boulder, USA, Hurrell [2003, updated regularly; accessed 19th March 2018]). The monthly NAO indicated that in 2010 the December was particularly harsh. At this time of the winter, Natterer's bats are typically still active as indicated by a lot of activity at the hibernaculum according to the logger data (own unpublished data), and especially males try to accumulate their fat reserves very late in Natterer's bats (Kohyt, Rozik, Kozakiewicz, Pereswiet‐Soltan, & Gubała, 2016). In contrast, Daubenton's bats start to hibernate much earlier (own unpublished data) and thus should not have been affected by the cold conditions during that month. Winter 2012/2013 showed an even lower NAO value than winter 2010/2011 but unlike in the winter 2010/2011 in winter 2012/2013, the lowest NAO value was in March, the month when individuals of both species depart from the hibernaculum (own unpublished data). Thus, if bats die during that period, in our analyses, we would find a low survival for the summer 2013. This is the case in both species with the lowest estimated summer survival in summer 2013 in adults of both species (mn: 0.78 (*SE* ± 0.03); md: 0.69 (*SE* ± 0.01)). In conclusion, adverse weather conditions at different seasons might result in species‐specific responses, depending on their respective foraging strategy and hibernation phenology.

After excluding the winter 2010/2011 with an exceptional population crash in Natterer's bats, the data revealed a stable seasonal survival pattern that varied in severity across years for adult bats in both species. Even though our study species are both of small body size, winter survival was significantly higher than summer survival in both of them. This is in contrast to the concept of summer‐ and winter‐regulated bird populations (Blumstein & Fernández‐Juricic, 2010; Newton, 1998) but it is in line with what has been described for mammalian hibernators (Turbill et al., 2011). The difference in seasonal survival in adult Natterer's bats (estimated summer survival: 0.78–0.86; estimated winter survival: 0.91–0.95) was smaller than the respective difference in adult Daubenton's bats (estimated summer survival: 0.69–0.84; estimated winter survival: 0.95–0.98). Adult Daubenton's bats had slightly lower survival rates during summer and higher survival rates during winter, than adult Natterer's bats each year. This is in line with our prediction with regard to their different foraging strategies and activity patterns during the hibernation period (Table S6). The consistently switching seasonal pattern when comparing both species (adult survival in winter: mn < md; in summer: mn > md) might indicate a different season‐specific sensibility of the two species to unfavorable conditions. The population crash of Natterer's bats occurring in a winter (2010/2011) and the lowest survival probabilities in Daubenton's bats occurring during two summers (2012 and 2013; Figure 1), additionally, suggests a species‐specific sensibility to the timing of unfavorable conditions.

### 4.2 Importance of individual characteristics for survival in the two species

In contrast to other studies (Culina et al., 2017; Monticelli et al., 2014), we found no consistent difference in survival between sexes in either species. For comparison, Culina and co‐authors reported a generally higher survival of females in Natterer's bats. The authors suggested higher costs for male Natterer's bats due to the polygamous mating system and, thus, a higher intensity on sexual selection for males (Culina et al., 2017). If this is the case, it cannot be confirmed in our study populations.

Finally, juvenile survival rates were lower than adult survival rates in both species. This confirmed results of other studies investigating age class‐specific survival in bats (Culina et al., 2017; Sendor & Simon, 2003) and underlines the importance of adult survival in long‐lived species, including bats, for population stability (Fleischer et al., 2017; Gaillard & Yoccoz, 2003; Péron et al., 2016). The difference between juvenile and adult survival was more pronounced in Daubenton's bats than Natterer's bats. Compared with other studies, however, the reduction in survival in juveniles was moderate in our study. Culina and co‐authors report a juvenile survival probability over winter of about 0.4 in Daubenton's bats (Culina et al., 2017), whereas in our study it was estimated to be about 0.66. Potential explanations for this high difference in juvenile winter survival in the two studies might be either population‐specific effects on juvenile survival or differences in the methods of analyses. Culina et al. (2017) investigated winter survival based on summer roosts and we investigated seasonal survival at a hibernaculum. Our estimated rates for juvenile winter survival might have been higher than the rates found by Culina et al. (2017) because we only included those juveniles that arrived at the hibernaculum. Thus, our estimates did not consider the juvenile stage between becoming volant and first migration to the wintering site which presumably are associated with a high mortality risk. To distinguish between those possible causes, individual‐based long‐term data sets monitoring individual bats over their life time at their summer roosts and hibernaculum are required.

## 5 CONCLUSIONS

With the exception of the exceptional winter 2010/2011 in Natterer's bats, our data revealed a stable seasonal difference in adult survival which varied in its severity across years in both species. Nevertheless, our data emphasize the importance of specific periods as potential drivers of population dynamics and suggest that at least in bats responses to global climate change might be species‐, population‐, and even age class‐specific. Environmental conditions that are advantageous for one species, population, or age class might be detrimental to others. Especially, the timing of adverse weather events might play an important role and needs to be further investigated in future studies in order to better understand population responses to global climate change. Clearly, this will also be a prerequisite for conservation.

## CONFLICT OF INTEREST

We have no competing interest.

## AUTHOR'S CONTRIBUTIONS

AS, CR, JG, and GK designed and conceived the study. LG and FM collected the field data. CR prepared the data for analysis. CR and JG carried out the statistical analysis. All authors interpreted the results, wrote the manuscript, and gave final approval for publication.

## Data Availability

Data available from the Dryad Digital Repository: https://doi.org/10.5061/dryad.b107q48.
