# Supplementary material for: Differences in seasonal survival suggest species‐specific reactions to climate change in two sympatric bat species
Source: Ecol Evol. 2019 Jul 2;9(14):7957–65. doi: 10.1002/ece3.5292 (PMC6662409; doi:10.1002/ece3.5292)
Supplement: Supplementary file 1 [file ECE3-9-7957-s001.docx]

**Supporting information (S): Differences in seasonal survival suggest species-specific reactions to climate change in two sympatric bat species**

**Tables**

Table S1 Description of the annual timing of trapping at the hibernaculum ‘Brunnen Meyer’ during the study period (2010/11 – 2014/2015). The time period of trapping and the number of trapping events are given.

| **Year** | **Timing of trapping events** | **Number of trapping events** |
| --- | --- | --- |
| 2010 | 15^th^ August – 28^th^ September | 16 |
| 2011 | 15^th^ August – 30^th^ September | 11 |
| 2012 | 23^th^ August – 25^th^ September | 7 |
| 2013 | 23^th^ August – 1^st^ October | 7 |
| 2014 | 26^th^ August – 30^th^ September | 8 |

Table S2 Descriptive analyses regarding the number (nr) of individuals (ind) per species (Natterer's bats (*Myotis nattereri*, mn) and Daubenton's bats (*Myotis daubentonii*, md)) that have been missing at least in one arrival or emergence period and were excluded from the data set in the survival analyses.

| Parameter | Natterer’s bats | Daubenton’s bats |
| --- | --- | --- |
| Total number of individuals | | |
| Nr ind missing / nr ind total | 62 / 820 | 24 / 625 |
| % ind missing | 7.56 | 3.84 |
| Of the missing individuals | | |
| % were missing once | 82.26 | 87.50 |
| % were missing twice | 12.90 | 12.50 |
| % were missing ≥ 3 | 4.84 | 0.00 |
| Of the missing events | | |
| % events were missing arrivals | 26.92 | 44.44 |
| % events were missing emergences | 73.08 | 55.56 |

Table S3 Number of individuals per time period and species, Natterer‘s bats (*Myotis nattereri*, mn) and Daubenton’s bats (*Myotis daubentonii*, md) assigned according to sex and age class (juv – juveniles; ad - adults). Winter periods are denoted by ‘w’ and the respective years, while summer periods are labelled as ‘s’ combined with the respective year. Due to sampling at a hibernaculum, we were only able to assign juveniles in winter.

| **Time Period** | **Natterer’s bats** | | | | **Daubenton’s bats** | | | |
| --- | --- | --- | --- | --- | --- | --- | --- | --- |
|  | **Male** | | **Female** | | **Male** | | **Female** | |
|  | **Juv** | **Ad** | **Juv** | **Ad** | **Juv** | **Ad** | **Juv** | **Ad** |
| w 2010/11 | 72 | 55 | 41 | 53 | 35 | 17 | 27 | 50 |
| s 2011 | / | 35 | / | 46 | / | 32 | / | 64 |
| w 2011/12 | 58 | 53 | 33 | 67 | 32 | 71 | 29 | 90 |
| s 2012 | / | 85 | / | 88 | / | 91 | / | 100 |
| w 2012/13 | 45 | 93 | 34 | 99 | 17 | 104 | 15 | 88 |
| s 2013 | / | 112 | / | 120 | / | 106 | / | 98 |
| w 2013/14 | 60 | 107 | 24 | 128 | 32 | 124 | 16 | 97 |
| s 2014 | / | 144 | / | 136 | / | 139 | / | 109 |
| w 2014/15 | 31 | 146 | 38 | 160 | 29 | 165 | 21 | 115 |
| s 2015 | / | 159 | / | 175 | / | 176 | / | 131 |

**Table S4** Estimated survival probabilities of Natterer’s bats (*Myotis nattereri*, mn) and Daubenton’s bats (*Myotis daubentonii*, md). Winter periods are denoted by “w” combined with the respective years, while summer periods are labelled as “s” and the respective year. “Dead” and “Survived” denote the numbers of individuals (adults and juveniles combined) that were considered dead or have survived the respective period. The following column gives the proportion of survivors (in %). In each period, the null hypothesis of equal survival in the two species was tested (two-sided alternative, chi-squared test with *df*=1, Yate’s continuity correction). The final two columns give the value of the test statistic and the resulting *p*-value. Significant results are printed in bold letters and significance level is indicated by stars (* <0.05, ** <0.01, ***<0.001). A tendency is characterized by italic letters and is indicated by a point (**^.^** < 0.1).

| **Period** | **Species** | **Dead** | **Survived** | **% survived** | **X²** | ***p*-value** |
| --- | --- | --- | --- | --- | --- | --- |
| **w 10/11** | **mn** | **140** | **81** | **36.7** | **44.980** | **1.99x10^-11^ ***** |
|  | **md** | **33** | **96** | **74.4** |  |  |
| s 11 | mn | 17 | 64 | 79.5 | 0.084 | 0.772 |
|  | md | 23 | 73 | 76.0 |  |  |
| w 11/12 | mn | 38 | 173 | 82.3 | 1.037 | 0.309 |
|  | md | 31 | 191 | 86.0 |  |  |
| **s 12** | **mn** | **23** | **150** | **87.3** | **7.978** | **0.005 **** |
|  | **md** | **49** | **142** | **74.3** |  |  |
| *w 12/13* | *mn* | *39* | *232* | *86.0* | *2.985* | *0.084* ***^.^*** |
|  | *md* | *20* | *204* | *91.1* |  |  |
| s 13 | mn | 57 | 175 | 75.0 | 1.306 | 0.253 |
|  | md | 61 | 143 | 70.1 |  |  |
| w 13/14 | mn | 39 | 280 | 88.0 | 2.647 | 0.104 |
|  | md | 21 | 248 | 92.2 |  |  |
| s 14 | mn | 30 | 250 | 89.1 | 0.415 | 0.519 |
|  | md | 32 | 216 | 87.1 |  |  |
| w 14/15 | mn | 41 | 334 | 89.2 | 2.390 | 0.122 |
|  | md | 24 | 306 | 92.7 |  |  |
| s 15 | mn | 54 | 280 | 83.4 | 1.751 | 0.186 |
|  | md | 63 | 244 | 79.5 |  |  |

Table S5 Estimates for the mixed-effects logistic regression model with survival as response for Daubenton’s bat (*Myotis daubentonii*, md) and Natterer’s bat (*Myotis nattereri*, mn; excluding winter 2010/11). Winter periods are denoted by “w” and the respective years, while summer periods are labelled as “s” combined with the respective year. A random intercept controls for repeated observations of the same individual across years. The number of observations (N_observations_) and the number of individuals (N_groups_) that were included in the model are given. Estimates are the regression parameters on the logit scale. In parentheses, we give 95% confidence interval of each estimate. The parameter estimates for the variable year give deviations from the mean (sum contrasts). The parameters for season and age class are relative to the reference category. The resulting intra-individual correlation is ICC=(0.9²)/((0.9²)+(pi²/3))= 0.19 for mn and md.

| Fixed effects | Estimates (95% CI) | Additional model information | Estimates  (95% CI) |
| --- | --- | --- | --- |
| Natterer’s bats (surv ~ age class + season + year + (1\|ID)) | | | |
| Intercept | 1.6 (1.4;1.8) | Random intercept standard deviation | 0.9 (0.5;1.3) |
| Age status “juvenile” (Ref=”adult”) | -1.6 (-2.0;-1.2) |  |  |
| Season “winter” (Ref=”summer”) | 1.1 (0.7;1.4) | Data subset | s2011 – s2015 |
| Year “2010_2011” (Ref=”mean”) | -0.1 (-0.6;0.4) |  |  |
| Year “2011_2012” (Ref=”mean”) | 0.2 (-0.1;0.5) |  |  |
| Year “2012_2013” (Ref=”mean”) | -0.3 (-0.6;0.0) |  |  |
| Year “2013_2014” (Ref=”mean”) | 0.3 (0.0;0.5) | N_observations_ | 2276 |
| Year “2014_2015” (Ref=”mean”) | 0.0 (-0.3;0.2) | N_groups_ | 618 |
| Daubenton’ bats (surv ~ age class + season + year + (1\|ID)) | | | |
| Intercept | 1.2 (1.0;1.3) | Random intercept standard deviation | 0.9 (0.5;1.2) |
| Age status “juvenile” (Ref=”adult”) | -2.6 (-3.0;-2.1) |  |  |
| Season “winter” (Ref=”summer”) | 2.1 (1.7;2.5) | Data subset | w2010/11 – s2015 |
| Year “2010_2011” (Ref=”mean”) | -0.2 (-0.5;0.1) |  |  |
| Year “2011_2012” (Ref=”mean”) | -0.2 (-0.4;0.1) |  |  |
| Year “2012_2013” (Ref=”mean”) | -0.3 (-0.6;-0.1) |  |  |
| Year “2013_2014” (Ref=”mean”) | 0.5 (0.3;0.8) | N_observations_ | 2220 |
| Year “2014_2015” (Ref=”mean”) | 0.2 (-0.1;0.4) | N_groups_ | 601 |

Table S6 Summary of the individual median number of recorded days (med nr rec days_i_), separately for the arrival periods and the emergence periods in Natterer’s bats (*Myotis nattereri*) and Daubenton’s bats (*Myotis daubentonii*).

| **Species** | **Median med nr rec days_i_ arrival (min; max)** | **Median med rec days_i_ emergence (min; max)** |
| --- | --- | --- |
| Natterer’s bats | 7.0 (1.0; 60.0) | 1.5 (1.0; 12.0) |
| Daubenton’s bats | 2.3 (1.0; 16.0) | 1.0 (1.0; 7.0) |

**Figures**


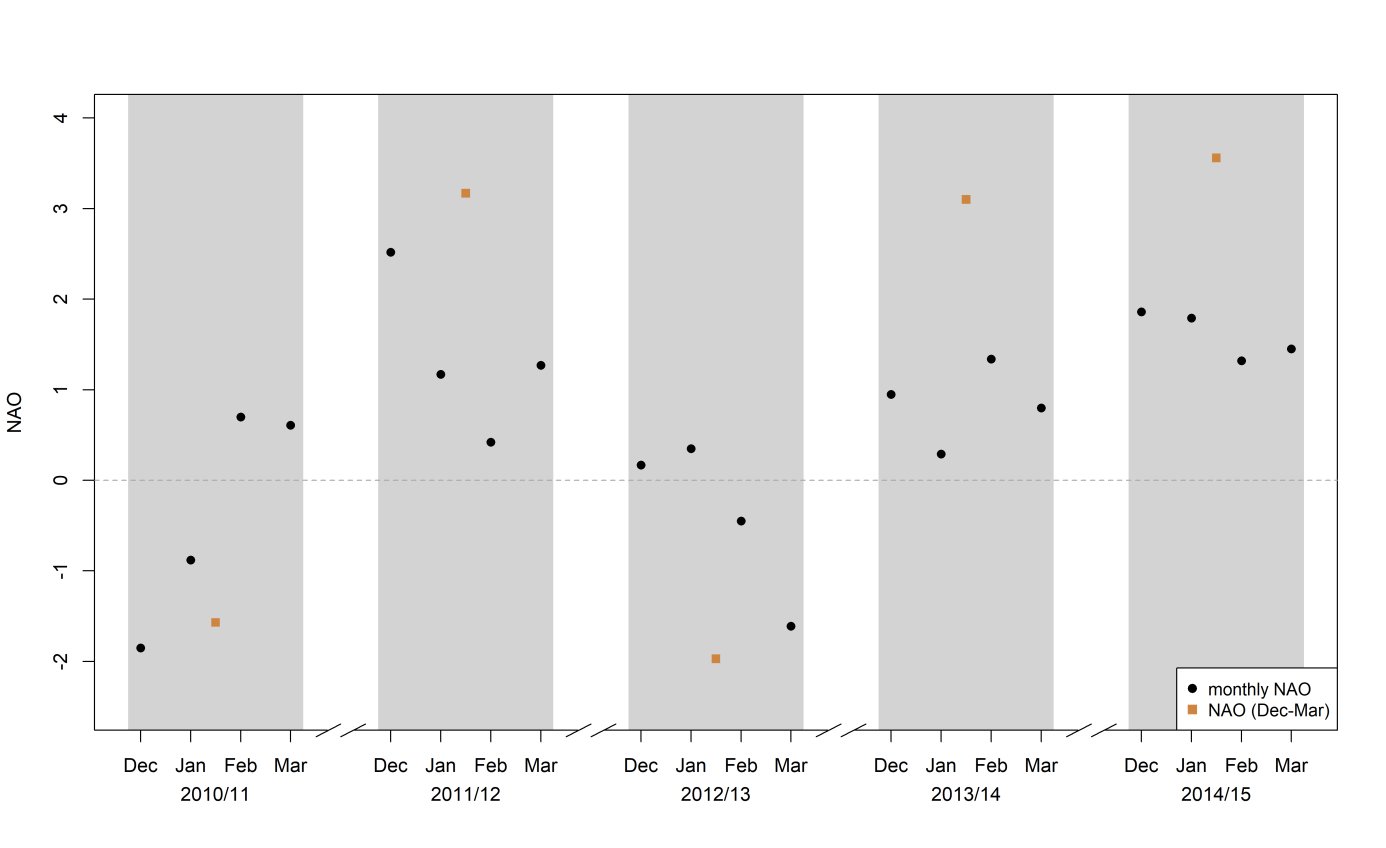


Figure S1 Description of the North Atlantic Oscillation index (NAO) during the study period (2010/11 - 2014/15). The NAO is based on the difference of normalized sea level pressure between Lisbon, Portugal and Reykjavik, Iceland (Hurrell 1995). It is described as a large-scaled weather parameter for winter severity (Hurrell 1995; Post et al. 1997). The dashed line highlights a NAO value of zero. Positive NAO values are associated with wetter/milder weather, while negative values are associated with dryer/colder weather over western Europe during winter (Hurrell, 1995). The grey background underlines the important winter months (December - March) for the calculation of the NAO. Black points represent the monthly NAO values and the orange squares characterizes the winter station-based index of the NAO (1^st^ of December until 31^st^ of March). NAO index data were provided by the Climate Analysis Section, NCAR, Boulder, USA, Hurrell (2003, updated regularly; accessed 19th March 2018).
